# The Role of Echocardiography in Neonates and Pediatric Patients on Extracorporeal Membrane Oxygenation

**DOI:** 10.3389/fped.2018.00297

**Published:** 2018-10-26

**Authors:** Carles Bautista-Rodriguez, Joan Sanchez-de-Toledo, Eduardo M. Da Cruz

**Affiliations:** ^1^Pediatric Cardiology Department, Hospital Sant Joan de Deu Barcelona, Universitat de Barcelona, Barcelona, Spain; ^2^Department of Paediatric Cardiology, Royal Brompton Hospital, London, United Kingdom; ^3^Division of Cardiac Intensive Care, Department of Critical Care Medicine, University of Pittsburgh, Pittsburgh, PA, United States; ^4^Department of Pediatrics, Heart Institute, Children's Hospital Colorado, School of Medicine, University of Colorado Denver, Aurora, CO, United States

**Keywords:** echocardiography, ECMO, pediatrics, neonatal, function assessment

## Abstract

Indications for extracorporeal membrane oxygenation (ECMO) and extracorporeal cardiopulmonary resuscitation (ECPR) are expanding, and echocardiography is a tool of utmost importance to assess safety, effectiveness and readiness for circuit initiation and separation. Echocardiography is key to anticipating complications and improving outcomes. Understanding the patient's as well as the ECMO circuit's anatomy and physiology is crucial prior to any ECMO echocardiographic evaluation. It is also vital to acknowledge that the utility of echocardiography in ECMO patients is not limited to the evaluation of cardiac function, and that clinical decisions should not be made exclusively upon echocardiographic findings. Though echocardiography has specific indications and applications, it also has limitations, characterized as: prior to and during cannulation, throughout the ECMO run, upon separation and after separation from the circuit. The use of specific and consistent echocardiographic protocols for patients on ECMO is recommended.

## Introduction

Extracorporeal membrane oxygenation (ECMO) and extracorporeal cardiopulmonary resuscitation (ECPR) provide temporary pulmonary and/or cardiac support when medical management fails (1, 2). Neonatal and pediatric research suggest that ECMO and ECPR provide a survival benefit (3–5), which has prompted the expansion of indications for ECMO and ECPR in both cardiac and non-cardiac patients.

Overall, neonatal ECMO utilization has significantly decreased over the last 25 years (6, 7). Neonatal respiratory diseases such as meconium aspiration syndrome, persistent pulmonary hypertension and congenital diaphragmatic hernia still account for the majority of neonatal cases requiring ECMO support (6). However, the need for ECMO support for these conditions has decreased dramatically due to significant improvement in medical management of hypoxemic respiratory failure in newborns, exogenous use of surfactant and use of inhaled nitric oxide (8–12). On the other hand, the number of neonatal cardiac patients requiring ECMO support has increased throughout the last two decades. Congenital heart defects represent the majority of neonatal cardiac cases in need of ECMO support (6). In contrast to neonatal ECMO usage, pediatric ECMO volumes have increased for both pulmonary and cardiac diagnoses. Congenital heart defects, non-ARDS acute respiratory failure and viral pneumonia account for the majority of pediatric cases requiring ECMO support (2, 6). ECPR in neonatal and pediatric patients has also become a valuable technique. There is strong evidence that ECPR provides a significant survival advantage over conventional cardiopulmonary resuscitation, and its use is becoming widespread (6, 13–15).

Over the years, the role of echocardiography in ECMO patients has transitioned from a primary means of monitoring cardiac function to a clinical tool whose findings are integrated into the overall evaluation and management of neonatal and pediatric ECMO patients. Echocardiography is a tool of utmost importance to assess indications for ECMO, choose the appropriate mode of support, and rule out mechanical complications such as anatomic defects (tamponade or aortic regurgitation) or ECMO-system complications (cannula position or thrombus). Echocardiography has also emerged as a risk stratification tool in the context of assessing ventricular function in order to determine the degree of potential ventricular recovery.

Despite all of this, some challenges remain, as final outcomes for veno-venous (VV) and veno-arterial (VA) ECMO have changed little over the past 10 years. Survival following pulmonary support remains around 80% in neonates (6, 7) and 55% in the pediatric population (2, 6). Survival after cardiac support is reported to range from 38 to 55% in neonates (16, 17) and around 55% in the pediatric population (2, 6).

This manuscript aims to provide an overview of key elements to consider in the echocardiographic evaluation of neonates and pediatric patients requiring ECMO. It highlights those areas where additional evidence is still needed and points out potential research strategies.

## Overview of ECMO and important physiological and hemodynamic changes

**Figure 1:**
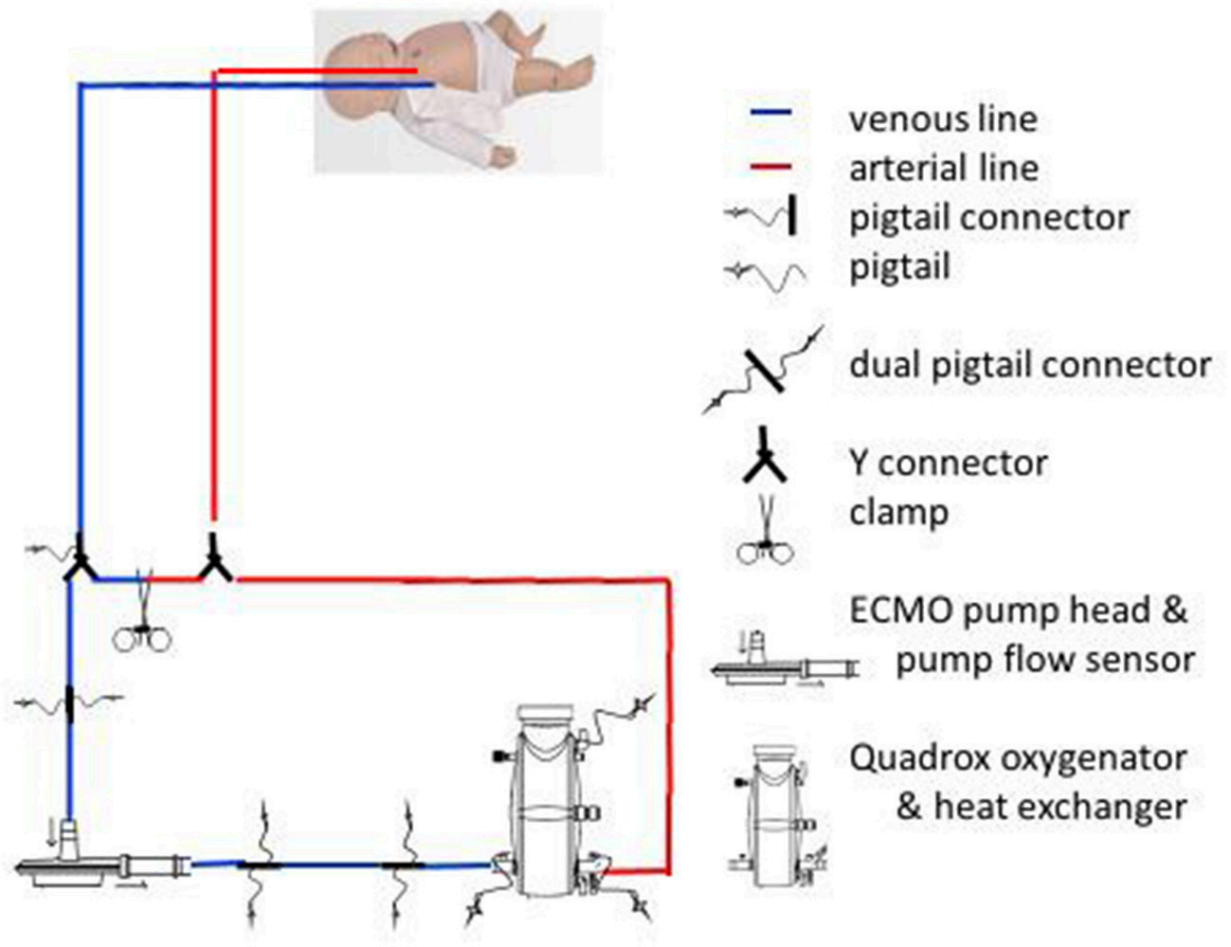
ECMO (VA) circuit anatomy.

Performing an accurate and clinically useful echocardiographic evaluation is mandatory in order to understand the ECMO circuit anatomy and physiology for each different modality (VV ECMO and VA ECMO). ECMO is a form of extracorporeal life support that evolved from cardiopulmonary by-pass. In ECMO, an artificial circulator carries venous blood from the patient to a gas exchange device (oxygenator). In the oxygenator, blood becomes enriched with oxygen and carbon dioxide is removed. This blood then re-enters the patient's circulation. Circuit flow is achieved using either a centrifugal or a roller pump (Figure 1), but the discussion of the technical and clinical applications does not pertain to the nature and scope of this review.

Patients who are hypoxemic despite maximum conventional ventilator support, have significant ventilator-induced lung injury, or are in cardiogenic shock may be considered for ECMO support. In all cases, the clinical situation of the patient should be considered highly treatable and reversible in order to be a candidate for ECMO. In those conditions with respiratory failure, ECMO will allow the level of ventilator support to be reduced and time for recovery from the underlying pathology and/or ventilator-induced lung injury. The same principle applies to ECMO for cardiogenic shock, allowing time for the myocardium to rest and recover. Regardless of the indication for ECMO, neonates and children without lung/myocardial recovery may require heart/lung transplantation. ECMO remains as one of the primary options for mechanical ventilation and/or circulatory support as a bridge to heart/lung transplant (18, 19).

### Types of ECMO

There are two primary types of ECMO: Veno-Venous (VV) and Veno-Arterial (VA).

VV ECMO provides pulmonary support with some degree of indirect cardiac support. VV ECMO improves the patient's oxygenation by reducing the amount of blood that passes through the lung without being oxygenated and removing CO_2_ from the blood. This allows the level of ventilator support to be decreased, which reduces ventilator-induced lung injury. The efficiency of oxygenation by the ECMO circuit depends on the pump flow relative to the patient's cardiac output. The patient's oxygenation should increase with an increasing ECMO flow rate. If this does not occur, it should raise suspicion of recirculation of blood between the inflow and outflow cannulas.

VV ECMO is more efficient at removing CO_2_ from the blood than delivering oxygen. The amount of CO_2_ removal depends on the ECMO flow rate relative to the patient's cardiac output as well as the oxygen flow rate relative to the oxygenator. Increasing the oxygen flow rate decreases the CO_2_ in the blood leaving the oxygenator (analogous to the effect that increasing minute ventilation has on arterial PCO_2_).

Arterial O_2_ is determined by the relationship between the ECMO pump flow and the patient's cardiac output. If the ECMO pump flow is low compared to patient's cardiac output, the arterial O_2_ will also be low. Increasing the ECMO pump flow will increase the O_2_ in the mixed venous circulation and therefore increase the arterial O_2._

VA ECMO provides direct cardiac and pulmonary support. In VA ECMO, deoxygenated blood is drained from the patient's venous circulation into the ECMO circuit. Blood is oxygenated via the oxygenator and is returned to the arterial circulation, providing support for both heart and lung failure. It can be used at low rates to provide partial assistance or at high rates to completely replace the patient's cardiac output. Therefore, PO_2_ levels in VA ECMO are the result of mixing between the ECMO flow and native cardiac output.

It is worth noting that in VA ECMO systems, any residual native cardiac output may pass through the lungs. If the lungs are affected by disease or the mechanical ventilation is insufficient, blood from the residual cardiac output will remain hypoxic as it enters the systemic circulation from the left ventricle (LV) in an anterograde fashion. In this clinical setting, manipulation of ECMO flow is indicated to reduce native cardiac output. Reducing ventilator support to rest settings, especially lowering the FiO_2_, is recommended to minimize hyperoxic injury and allow time for pulmonary and cardiac recovery (20–22). Of note, pulmonary venous return from bronchial arteries can also place the LV at risk of distension, particularly if there is severe LV dysfunction. Echocardiography plays a significant role in assessing and monitoring left heart cavity distension under VA-ECMO.

In both of these ECMO support modalities, it may become imperative to create an inter-atrial communication to unload the left-sided-heart, either surgically or by cardiac catheterization (23–25). This situation is rarely encountered in the neonatal setting, as the foramen ovale is commonly patent.

### Physiological effects of ECMO

VA-ECMO is hemodynamically distinct from VV-ECMO. In order to understand the hemodynamics, a review of venous return and cardiac function is warranted. With respect to the right heart, venous return is determined by the mean venous pressure. This pressure depends on the *volume status* of the patient's venous beds, the adrenergic tone and the resistance to venous return (26–28). Moreover, valvular competence, heart rate and rhythm, contractility, diastolic properties and afterload also contribute to venous return. All of these properties together are described by the Frank-Starling curve (29, 30).

In the left heart, venous pressure originates within the pulmonary circuit (31), and pulmonary venous return is mediated by the cardiac function of the left heart. As in the right heart, the slope of the cardiac function curve is determined not just by contractility, but also by multiple variables such as afterload. Thus, an increase in left heart afterload will decrease the slope of the cardiac function curve.

VV-ECMO decreases pulmonary vascular resistance, reduces right ventricle (RV) afterload and improves RV function due to the resolution of severe hypoxemia and hypercapnia. VA-ECMO has been associated with more physiological effects than VV-ECMO, as it short-circuits the normal hemodynamic pathway. When VA-ECMO is initiated, blood is removed from the venous bed and venous return to the right heart decreases. This causes a subsequent decrease in right atrial pressure, IVC volume and pulmonary blood flow, which will in turn affect gas exchange (32–34). After blood has gone through the VA-ECMO circuit, it is returned to the aorta. High arterial blood volumes will result in higher afterload to the LV. As a consequence, there is a reduction in cardiac output as well as a drop in left atrial pressure proportional to the increase in pulmonary venous return (34). As VA-ECMO is often initiated in patients with poor LV function, this effect may be profound. However, because pulmonary blood flow and pulmonary venous return are diminished, the rise in left atrial pressure is minimal. Nevertheless, acute pulmonary edema upon initiation of VA-ECMO has been described (35). In such patients, it may be beneficial to consider left atrial decompression (balloon atrial septostomy or placement of a new left atrial cannula) or transitioning to ventricular assist device [VAD] rather than ECMO (36).

## Echocardiography for patients on ECMO support

### Overview and limitations

Echocardiography plays a significant role in a patient supported with ECMO. It provides information regarding patient selection, guides the insertion and placement of cannulas, monitors progress, detects complications and recognizes recovery, readiness for weaning and ECMO decannulation. However, it is important to acknowledge that management decisions should not be made exclusively based upon echocardiographic findings.

The primary utility of echocardiography in this patient population relates to the evaluation of anatomy and residual lesions, cardiac function and trends, arrhythmias, and ECMO cannula position, flow and potential complications. Echocardiography has the advantages of being a non-invasive technique that is readily available at the patient's bedside and provides real-time evaluation of cardiac structure with high sensitivity and specificity (37–39).

This being said, echocardiography for ECMO patients is fraught by several limitations. Clinical correlation is essential and a *sine qua non* condition to be efficient. Patients on ECMO support are assessed by multiple observers, which may lead to subjective interpretation of clinical status. Moreover, physiological changes induced by ECMO may alter echocardiographic findings. A systematic adherence to the echocardiographic protocol and quantification of the echocardiographic parameters may help to avoid those biases. However, the published literature on the role of echocardiography in ECMO remains scarce (37, 40).

Position papers on the management of pediatric ECMO recommend that an echocardiography-trained physician should be part of the team caring for neonates and children on ECMO (*ELSO guidelines*) (15). However, echocardiography might be less available than chest X-ray and require a well-trained team with specific skills, which might have implications in management costs (41).

### Pre-ECMO echocardiography

Before initiating ECMO support, it is crucial to perform an echocardiographic assessment to identify the underlying cardiac anatomy, recognize the patient's hemodynamic condition and help to choose the best ECMO modality. VV-ECMO will provide support for severe respiratory failure when no major cardiac dysfunction exists, and VA-ECMO will provide support for severe cardiac failure most commonly after cardiac surgery (2, 13, 14, 33, 38, 42). Echocardiographic assessment will also depend on the stability of the patient. In the case of cardiac arrest, echocardiography should be focused on anatomical defects and reversible hemodynamic conditions such as cardiac tamponade (43, 44). A checklist for pre-ECMO echocardiography assessment is proposed in Table 1.

**Table 1:** Pre-ECMO echocardiographic assessment checklist.

**Detailed anatomic description**
Rule out effusion and/or tamponade physiology Evaluate peripheral vascular patency Demonstrate IVC-RA continuity Evaluate right SVC, existence of left SVC and bridging vein Assess any possible shunt at the atrial or ventricular septum Assess duct patency, direction of the shunt and gradient Evaluate any surgically correctible condition
**Assess signs of RV pressure or volume overload**
RV function assessment
- Systolic function assessment ° Fractional area change ° TAPSE ° Pulmonary artery acceleration time (PAAT) ° s' ° Strain and strain rate ° Myocardial acceleration during isovolumic contraction - Diastolic function assessment ° E/A ° E/e' ° Deceleration time ° IVC collapse ° Flow reversal in hepatic veins during atrial contraction
**Acquisition of LA and LV dimensions**
LV function assessment
- Systolic function assessment ° Simpson's EF ° Aortic VTI ° Mitral regurgitation dP/dt ° s' ° Strain and strain rate ° Myocardial acceleration during isovolumetric contraction - Diastolic function assessment ° E/A ° E/e' ° Deceleration time ° Flow reversal in pulmonary veins during atrial contraction
**Valvular assessment**
Rule out significant aortic regurgitation

#### Anatomical description

Echocardiography is of paramount importance to provide detailed anatomical description of the heart and the great vessels. It can demonstrate inferior vena cava (IVC) and superior vena cava (SVC) to right atrium (RA) continuity, which is important for cannula placement given that the right internal jugular vein and the right common carotid artery are the most common sites for pediatric peripheral ECMO cannulation (14). A complete vascular anatomical description will help to detect any potential barrier to cannulation, as it allows for the identification of thrombi, vessel stenosis or occlusion, aneurysms, persistent left SVC or anomalies of the bridging vein that might affect the cannulation process. Femoral vessels should also be assessed in older children, as those could serve as accessory indwelling points if required. Presence, direction and gradient of atrial and ductal shunts should also be assessed for their potential interference with the oxygenation and the flow of ECMO. The anatomical evaluation will also identify any surgically correctible condition.

#### RV and LV function assessment

The assessment of RV function and RV dimensions is important prior to initiating ECMO support, although the complex shape of the RV chamber makes it difficult to use geometric models for volume and function quantification, and the retrosternal position of the RV creates a barrier for ultrasound waves. The finding of RV dilatation pre-ECMO initiation has been associated with increased intensive care unit mortality (45, 46). The aim of RV function assessment is to estimate RV pressure and demonstrate the presence and extent of ventricular dysfunction. The most useful echocardiographic parameters to assess these conditions in children are the estimation of pulmonary artery pressure (PAP), assessment of ductal or atrial shunt, assessment of interventricular septum and LV shape, TAPSE, pulmonary artery acceleration time (PAAT) and strain.

It is well known that PAP can be estimated by measuring the TR jet maximum velocity by continuous wave (CW) spectral Doppler (47). However, this method relies on assumed RA pressure and preserved RV function. For this reason, invasive measurement of the RA pressure should be obtained to accurately estimate PAP (48, 49). Alternatively, the PAAT can serve as a reliable parameter and can be easily calculated from the PW spectral of the pulmonary artery. Moreover, its values are highly predictive of pulmonary hypertension. A PAAT <110 ms in children is suggestive of pulmonary hypertension. A ratio of PAAT/RVET (right ventricular ejection time) <0.31 also suggests pulmonary hypertension (50, 51). In some situations, the right side of the heart and its hemodynamic status will have to be assessed in a more subjective way. In this setting, the presence of a ductal and/or atrial shunt can provide a quick indication as to whether PA pressure is higher than, equal to, or lower than systemic pressure. Another important marker of increased RV pressure is flattening of the interventricular septum with motion of the septum toward the LV during end-systole (52). Measurements of transverse and longitudinal lengths in the four-chamber view of the RV can be compared to the relative size of the LV. An RV diastolic area equal to or greater than that of the LV may indicate RV enlargement (53).

RV systolic function assessment in this group of patients can be quite difficult. The fractional area change has been found to correlate with magnetic resonance-derived RV ejection fraction. A percentage of fractional area change >35% is considered normal in adults (54–56). More detailed and validated studies in children are lacking. Tricuspid annular plane systolic excursion (TAPSE) evaluates longitudinal RV function. Normative values for age are available for children (57, 58). TAPSE has been found to be especially useful in children with pulmonary arterial hypertension, where TAPSE <15 mm has been associated with a higher event rate (59, 60). Despite this, the main utility of TAPSE is for individual longitudinal follow-up. Tissue Doppler systolic velocity of the tricuspid annulus is another measure of longitudinal RV systolic performance. Normal reference values for the pediatric population are also available (61). A value <11.5 cm per second is associated with global RV dysfunction with an ejection fraction < 45% (62, 63).

Increasingly, the evaluation of strain and strain rate of the RV is being used to assist in the evaluation of RV function. Low RV strain values, especially in the apical and free wall regions, have been found to strongly correlate with mean pulmonary artery pressures, right ventricular size and function and mortality (64, 65). The myocardial acceleration during isovolumetric contraction is a promising load-independent measure of ventricular contractile function. This Doppler-derived index is measured as the ratio of systolic velocity to the time to peak systolic velocity. Isovolumetric acceleration is strongly associated with tricuspid annular plane systolic excursion and tricuspid myocardial systolic velocity (66).

The assessment of RV diastolic function should be included in the routine pre-ECMO echocardiogram, as diastolic dysfunction can precede declines in systolic function and appears to correlate with risk factors such as RV dilatation (67, 68). Pulsed wave Doppler measurement of tricuspid inflow and myocardial DTI is one method of diastolic analysis that yields objective data for E/A and E/e' ratios. With normal diastolic function, the early filling velocity is higher than the atrial contraction velocity and, therefore, reversal of the E/A ratio < 0.8 with increased deceleration time represents impaired ventricular relaxation. Increased E/e' ratios >15 represent increased RV filling pressures (69, 70). Also, RV diastolic dysfunction can result in increased right atrial pressures that can cause decreased or absent collapse of the IVC and reversal of flow with atrial contraction in the hepatic veins (49).

Echocardiography assessment of LV function, dimension together with the analysis of the left atrium (LA) is paramount. LV ejection fraction by Simpson's method and aortic VTI should be calculated. These values should be interpreted clinically, considering inotropic drug doses being administered as well as ventilator settings. If mitral regurgitation is present, dP/dt should be estimated as a good surrogate of LV function (71, 72). Tissue Doppler values and Doppler flow parameters (as described for the RV) should be acquired to study both systolic and diastolic function. The estimation of LV function before ECMO implantation should guide the administration of inotropes and fluids needed during the procedure. LA size and function should also be assessed prior to initiating ECMO, as these values may be very important when deciding whether or not the LV or LA needs to be decompressed. Although there are no published data on the best cardiac function parameters while on ECMO, LA size, and function can be very reliable when assessing the pre-ECMO hemodynamic condition and used for comparison during the ECMO run and weaning.

Attention should always be payed to the ventricular interdependence phenomenon understanding that LV contraction directly impacts RV systolic function and vice versa (73, 74).

#### Assessment of valves

Valvular function should be assessed prior to ECMO initiation, because after cannula positioning, function cannot be correctly quantified due to ECMO flows. Pre-ECMO echocardiography can help to identify hemodynamic lesions that could explain the hemodynamic or respiratory compromise.

Aortic valve integrity is of paramount importance in patients who are progressing toward VA-ECMO support, as the arterial cannula will eject its flow into the aortic arch. Aortic valve regurgitation may contribute to LV distension, LV dysfunction and subsequently pulmonary hypertension (75–77).

Valvular dysfunction should be frequently re-evaluated during ECMO support, especially during weaning. For example, the detection of moderate mitral regurgitation before ECMO initiation should prompt close monitoring of fluid status, as it may be associated with increased pulmonary edema. If mitral prolapse preexists, repeated echocardiographic examinations during prolonged VV-ECMO support should be performed to exclude the development of endocarditis. Tricuspid valve anomalies should also be evaluated. The presence of tricuspid stenosis may compromise the flow of oxygenated blood from the RA to the RV. Severe tricuspid regurgitation may lead to increased RA pressure, and the consequent right to left shunt at the atrial level will manifest as severe hypoxemia. Pulmonary hypertension could be due to a preexisting condition of unknown chronic obstructive pulmonary disease or to a subacute undiagnosed pulmonary embolism.

### Echocardiography during cannulation

Echocardiography guidance and monitoring during ECMO cannulation in neonatal and pediatric patients is warranted (78, 79). Transthoracic echocardiography may not always provide the required spatial resolution, therefore transesophageal echocardiography (TEE) may be required (37, 80). There are 3 main goals in this setting: (1) to choose the appropriate cannula size, (2) to ensure the optimal placement of the cannula, and (3) to reduce the rate of complications associated with cannulation.

The size of the venous cannula is a major determinant of blood flow in the ECMO circuit. Therefore, the goal is to insert the largest possible cannula. Measuring the diameter facilitates the selection of the appropriate venous cannula size. Some authors suggest the cannula should be two thirds of the diameter of the vessel, others state that the size (in French) should be three times the diameter (in mm) or the same size as the circumference (in mm) of the vessel (81, 82). The arterial cannula size is usually smaller. In order to ensure adequate distal perfusion in femoral artery cannulation the placement of an arterial bridge is recommended (81, 83).

In neonatal and pediatric patients, echocardiography has been shown to be a better imaging modality to identify the correct placement of the cannula and to reduce the rate of surgical repositioning of ECMO catheters (79, 84–86). VV-ECMO can be achieved with either two cannulas or with one dual-lumen cannula. In the case of two-cannula VV-ECMO, the drain cannula tip is usually placed in the proximal IVC past the hepatic veins. When the cannula is positioned too distal in the IVC the cannula tip might end up against the vessel wall. In the other hand, if it is placed too proximally, there is a risk of damage to the interatrial septum or the tricuspid valve. The return cannula is usually inserted into the internal jugular vein and advanced through the SVC to the mid-right atrium. If the two tips are located too close together, recirculation may occur, resulting in hypoxemia (87, 88).

**Figure 2:**
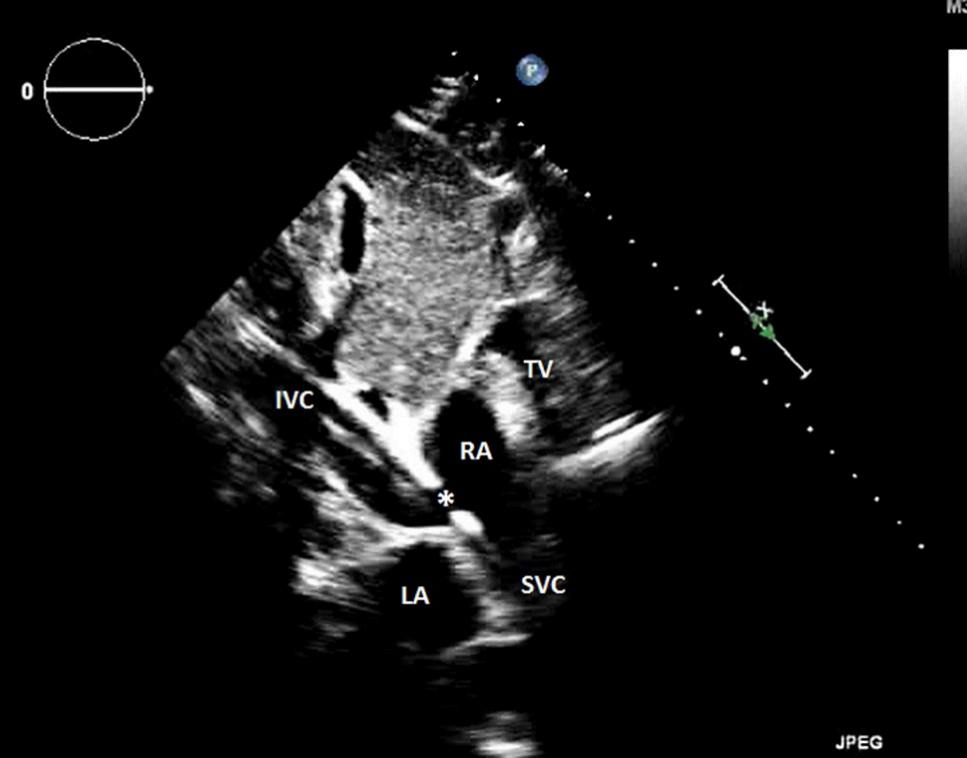
Transthoracic echocardiography showing an appropriate veno-venous cannula positioning, and B a venous cannula inserted too far into the inferior vena cava. RA, right atrium; LA, left atrium; IVC, inferior vena cava; SVC, superior vena cava; TV, tricuspid valve; *outflow orifice toward the tricuspid valve.

**Figure 3:**
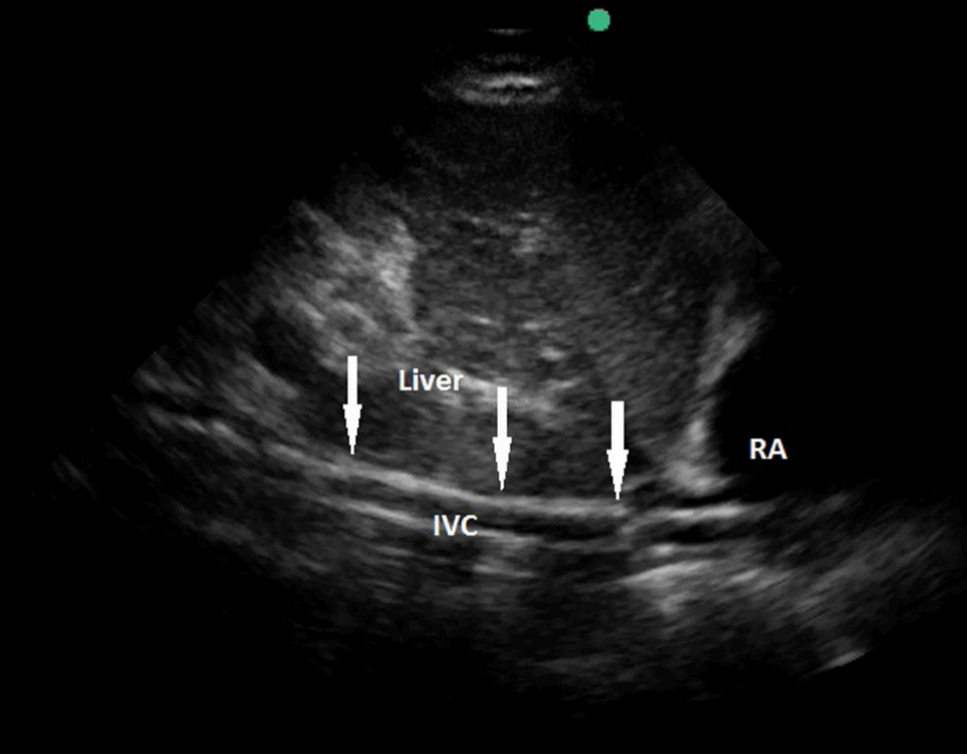
Transthoracic echocardiography showing a venous cannula inserted too far into the inferior vena cava. RA, right atrium; IVC, inferior vena cava; arrows pointing the course of the cannula.

In the case of single dual-lumen cannula VV-ECMO, the cannula is inserted into the SVC via the right internal jugular vein (Figures 2, 3) (80, 89). The drainage orifices of the cannula should be located at the levels of the SVC and IVC to allow for the removal of deoxygenated blood. The infusion port should be in the mid-right atrium and directed toward the tricuspid valve in order to deliver oxygenated blood directly to the RV (37, 80, 90–92). The dual-lumen cannula technique has the advantage of single-site cannulation and a lower probability of recirculation (88, 93). After cannulation, SVC and IVC venous inflow to the cannula as well as the infusion port position directed toward the tricuspid valve should be confirmed. Of note, the use of single dual-lumen cannulas has been banned in neonates due to the high occurrence of cardiac perforation (94, 95).

In peripheral VA-ECMO, the venous drainage is similar to that in VV-ECMO, with the drainage cannula positioned at mid-right atrium. In this case, the same precautions described for VV-ECMO should be taken. The arterial cannula is usually inserted through the right carotid, with the tip at the right brachiocephalic artery—aortic arch junction (85, 86). A direct jet to the aortic valve should be avoided. If necessary due to patient's weight, an additional arterial cannula may be placed thorough the femoral artery, with the tip sitting in the iliac artery or distal aorta. In any case, prompt detection of distal ischemic signs is warranted (83). In central VA-ECMO, the cannula is typically inserted directly into the patient's ascending aorta.

Echocardiography allows for real-time guidance of the cannulation process and helps to prevent procedure related complications such as vascular injury, hematoma, cardiac tamponade, ischemia, tricuspid valve damage, or atrial septum perforation (82, 96). Incorrect cannula positioning may require intervention or manipulation to achieve an appropriate location, thus increasing the risk for bleeding and infection (37, 84, 85, 97).

### Echocardiography during ECMO support

Echocardiography assessment should be routinely performed in patients on ECMO support and not only in the presence of hemodynamic instability. The clinical targets are summarized in Table 2.

**Table 2:** Clinical targets to be assessed during ECMO support.

**Cannula position**		
**Ventricular function**		
	**Right ventricle**	**Left ventricle**
Systolic function parameters	FAC TAPSE s' Strain and strain rate Myocardial acceleration during isovolumetric contraction	Size EF Simpson's method dP/dt (mitral regurgitation) Ao VTI s' Strain and strain rate Myocardial acceleration during isovolumetric contraction
Diastolic function parameters	E/A E/e' Deceleration time IVC collapse Flow reversal in hepatic veins	E/A E/e' Deceleration time Pulmonary veins flow pattern
**Complications**		
- Pericardial effusion		
- Thrombosis		
- Stunning		
- Decompressing		
- RV failure		

The correct positioning of the cannulas should be routinely monitored. Should the position of the cannulas are too superficial, an increased risk for incidental decannulation exists (90, 98). In cases where the venous cannula is too distal, there is an increased risk of venous return obstruction and ECMO malfunction. When the arterial cannula is too distally into the ascending aorta, it may cause aortic valve lesions and increase the LV afterload, leading to coronary hypoperfusion. According to the ELSO Registry (2), cannula malposition increases mortality and morbidity, accounting for a reported 11% of complications in newborns and 15% of complications in infants. Moreover, survival rates decrease from 85 to 68% in newborns and from 64 to 51% in infants due to cannula-related problems. For this reason, cannula position control and follow-up is mandatory to ensure optimal circuit performance, adequate venous unloading, minimal recirculation and prevention of anatomic obstruction.

Cardiac function in both the right and left ventricles should be carefully monitored during ECMO support. The effect of ECMO flow rates on changes in cardiac parameters has been described in the adult literature (99). However, data on how to echocardiographycally assess the ventricular performance in pediatric and neonatal ECMO is still scant. Therefore, measurements obtained prior to ECMO initiation can be compared to those obtained while on ECMO support. This parameter comparison becomes of utmost importance when initiating suitable treatments. For instance, the persistence of RV dysfunction despite ECMO support should trigger the adoption of rescue therapies such as inhaled nitric oxide. Moreover, the previously mentioned comparison might be useful to identify any changes in cardiac function when lowering ECMO flows. For example, as mentioned in the introduction, VV-ECMO does not change RV preload, but it increases the mixed venous oxygen saturation. This will cause a decrease in pulmonary vascular resistance as well as RV afterload. Due to RV-LV interdependence, this may indirectly improve LV function by increasing oxygen delivery to the coronary arterial circulation. All these changes can be fully monitored by means of the systolic and diastolic parameters mentioned in Table 2, and they can help to influence clinical decision-making.

**Figure 4:**
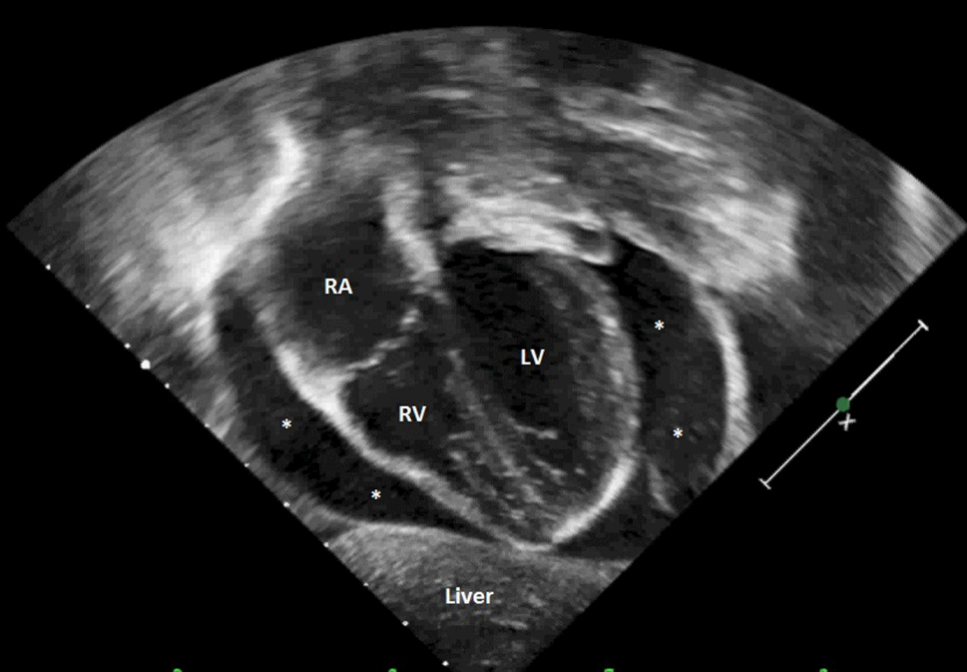
Transthoracic echocardiography showing severe cardiac tamponade. RA, right atrium; RV, right ventricle; LV, left ventricle; *severe pericardial effusion.

Echocardiography can help to detect complications during the ECMO course. Patients on ECMO support are more susceptible to bleeding due to systemic anticoagulation, which may contribute to pericardial effusion and tamponade (Figure 4). Clinical diagnosis of tamponade in these patients remains challenging, especially in VA-ECMO. For this reason, echocardiography is paramount for identification and follow-up of any diagnosed effusion (100–102).

ECMO flow problems may occur both on VV and VA-ECMO. Low flow may be related to inadequate patient or cannula positioning and hypovolemia, but it is always important to rule out the presence of an intracannula thrombus (103–105).

**Figure 5:**
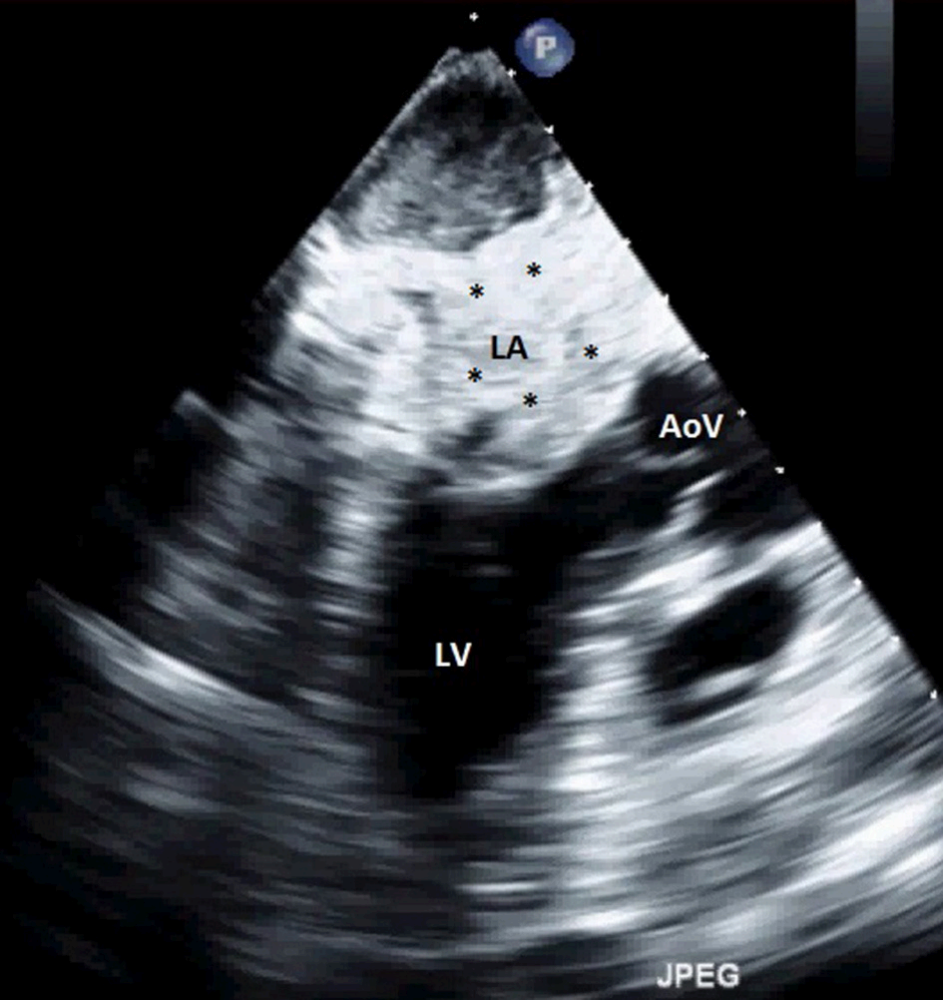
Transthoracic echocardiography showing an extensive LA thrombus. LA, left atrium; LV, left ventricle; AoV, aortic valve; *thrombus.

**Figure 6:**
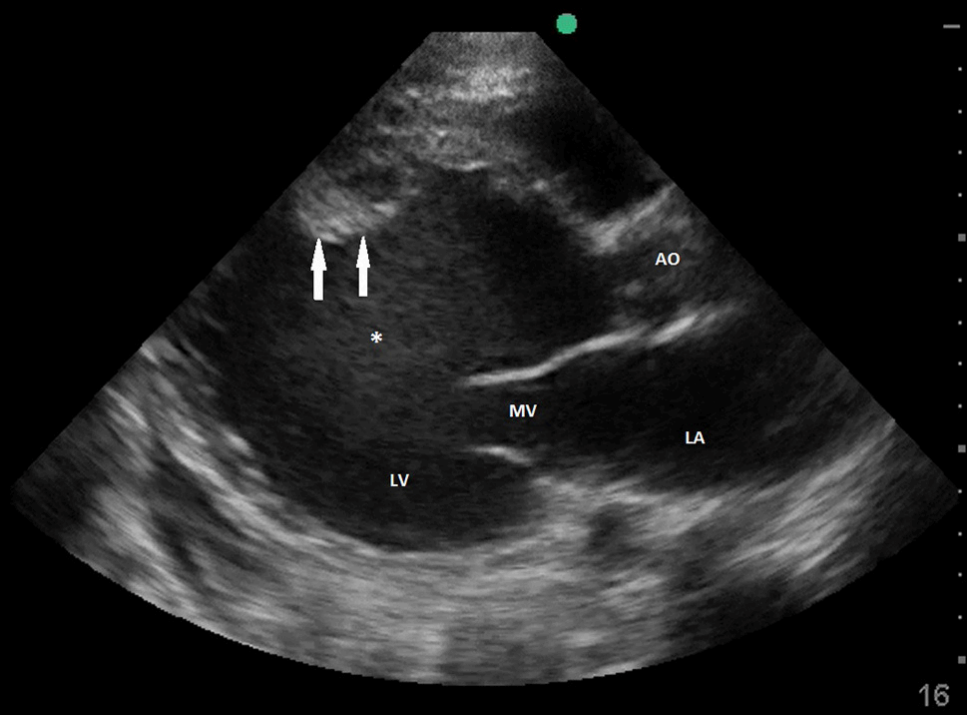
Transthoracic echocardiography showing severely dilated LV with spontaneous contrast and mural thrombus. LA, left atrium; MV, mitral valve; LV, left ventricle; Ao, Ascending aorta; *spontaneous contrast; arrows pointing mural thrombus.

Echocardiography can help monitor cardiac chamber size to ensure adequate emptying of the cardiac chambers. In this assessment, aortic valve opening is paramount. As described herein, VA-ECMO may compete with the stroke volume ejected from the LV. Any condition that leads to an increased LV afterload will ultimately cause LV distention, LV subendocardial ischemia, intracavitary thrombus formation (Figure 5) and pulmonary edema (106–109) that will delay cardiac function recovery (110). Presence of spontaneous echo contrast is a sign of this clinical setting (Figure 6) (111). It is important to remember that pulmonary venous return from bronchial arteries or major aorto-pulmonary collaterals can also place the LV at risk of distension. This risk is especially heightened during VA-ECMO, as the continuous blood return to the LA is not vented. In this clinical condition, reducing vascular resistance and ECMO flow rate can help to lower LV afterload and allow aortic valve opening. LV decompression has been recommended in case of LA and LV dilatation with associated risk for pulmonary edema (23, 112). Several techniques have been described for LV decompression, such as a septostomy (113, 114) or the insertion of a microaxial pump (115, 116).

### Echocardiography during the ECMO wean

The last step during ECMO management is to decide when to wean and separate from the circuit. The prediction of successful weaning has long been an area of active research. Successful ECMO weaning depends on several clinical, hemodynamic and echocardiographic variables. Therefore, ECMO weaning should be a decision based upon a comprehensive patient evaluation. Though echocardiography will not provide all the answers, it is a useful assessment tool.

In VV-ECMO, weaning should be fundamentally based on the degree of recovery of lung function. The evaluation of oxygenation and pulmonary compliance by decreasing the gas flow through the ECMO circuit is paramount. Contrasting from VA-ECMO weaning, the role of echocardiography in weaning from VV-ECMO is not clear at present, and to date, echocardiographic data during VV-ECMO weaning are lacking. However, it is fair to state that echocardiography becomes crucial to assess optimal RV function and the absence or degree of pulmonary hypertension. Normal parameters are strongly desirable to achieve successful weaning in this group of patients. Considerations for VV-ECMO weaning may include a significant decrease in PAP, estimated by TR jet (as long as RA pressure is invasively monitored), no right-to-left shunt at the atrial and/or ductal level, normal interventricular septum motion, TAPSE>11.5 mm, PAAT>110 ms and E/e' in the left free wall <15.

In VA-ECMO, echocardiography may play a more significant role. Studies have shown that patients who were successfully weaned from VA-ECMO were those with more stable hemodynamics during ECMO support reduction (99). Therefore, readiness for ECMO discontinuation should be based on the degree of LV and global function recovery, encompassed by a sufficient hemodynamic performance and adequate tissue perfusion markers. Some parameters have been suggested in adults with ventricular assist devices, such as an LV ejection fraction >20–25% in some studies (117–119) and >35–40% in others (120, 121), an LV outflow tract velocity-time integral >10 cm (122), absence of LV dilatation, a lateral mitral annulus peak systolic velocity >6 cm/s and no cardiac tamponade (117–121, 123). Overall, successful VA-ECMO weaning should be expected when LV ejection fraction, lateral E/Ea ratio, velocity-time integral, lateral strain and strain rate will all increase while systematically decreasing the level of mechanical support (99). Aissaoui et al. have also described ventricular interdependence as a strong negative prognostic value in adult patients assisted by ECMO due to cardiogenic shock (124).

Although recent research has started to evaluate how to assess the changes in echocardiographic indices during ECMO, there is still a lack of evidence and validation of the previous named parameters in the clinical setting of pediatric VA-ECMO.

## Conclusions

Echocardiography is undeniably a critical tool for the evaluation of patients on ECMO or progressing toward mechanical assistance. Its utility is apparent when evaluating potential ECMO candidates, preparing for cannulation, assessing readiness for separation from the circuit, and following-up after decannulation. Notwithstanding this well-recognized fact, therapeutic and weaning decisions should not depend exclusively on echocardiographic findings, but rather on the patient's functional capacities and the markers of tissue perfusion and end-organ performance. Reliable and efficient interpretation of the echocardiographic findings in patients requiring, undergoing or recently separated from the ECMO device requires an in-depth understanding of both the patient's as well as the ECMO circuit's anatomy and physiology. The use of protocols for the sake of consistency, low variability in practice and effectiveness is highly recommended, as it fosters better outcomes and data tracking for quality and safety purposes.

## Author contributions

CB-R, JS-d-T, and ED conceived of the presented idea, developed the theory and performed the research, verified the results, and supervised this work. All authors discussed the results and contributed to the final manuscript.

### Conflict of interest statement

The authors declare that the research was conducted in the absence of any commercial or financial relationships that could be construed as a potential conflict of interest.
